# Established Principles and Emerging Concepts on the Interplay between Mitochondrial Physiology and *S*-(De)nitrosylation: Implications in Cancer and Neurodegeneration

**DOI:** 10.1155/2012/361872

**Published:** 2012-08-13

**Authors:** Giuseppina Di Giacomo, Salvatore Rizza, Costanza Montagna, Giuseppe Filomeni

**Affiliations:** ^1^Research Centre IRCCS San Raffaele Pisana, Via di Val Cannuta, 247, 00166 Rome, Italy; ^2^Department of Biology, University of Rome “Tor Vergata”, Via della Ricerca Scientifica, 00133 Rome, Italy

## Abstract

*S*-nitrosylation is a posttranslational modification of cysteine residues that has been frequently indicated as potential molecular mechanism governing cell response upon redox unbalance downstream of nitric oxide (over)production. In the last years, increased levels of *S*-nitrosothiols (SNOs) have been tightly associated with the onset of nitroxidative stress-based pathologies (e.g., cancer and neurodegeneration), conditions in which alterations of mitochondrial homeostasis and activation of cellular processes dependent on it have been reported as well. In this paper we aim at summarizing the current knowledge of mitochondria-related proteins undergoing *S*-nitrosylation and how this redox modification might impact on mitochondrial functions, whose impairment has been correlated to tumorigenesis and neuronal cell death. In particular, emphasis will be given to the possible, but still neglected implication of denitrosylation reactions in the modulation of mitochondrial SNOs and how they can affect mitochondrion-related cellular process, such as oxidative phosphorylation, mitochondrial dynamics, and mitophagy.

## 1. Introduction

Nitric oxide (NO) is a gaseous and membrane diffusible radical molecule generated by the NADPH-dependent enzyme NO synthase (NOS) from L-arginine and oxygen [1, 2]. Three are the major isoforms of NOS that have been so far identified, namely, neuronal and endothelial NOS (nNOS or NOS1 and eNOS or NOS3, resp.), which are constitutively active, and the cytokine-inducible NOS (iNOS or NOS2), mainly expressed in immune system to face host attack [3, 4]. The biochemical characterization of NO as new signaling molecule, as well as its implication in cardiovascular function earned Furchgott, Ignarro, and Murad the Nobel prize in Physiology or Medicine in 1998. In particular, they provided the most consistent lines of evidence that NO activates guanylyl cyclase by a direct binding to heme iron (Fe-nitrosylation) and induces cGMP-mediated signaling [5], thus regulating blood vessel tone [6], immune response [7], neurotransmission [8], and many other organic functions. NO can also react with other oxygen-derived radical and nonradical species (ROS), thus generating more dangerous reactive nitrogen species (RNS, e.g., peroxynitrite, ONOO^−^), which target proteins and irreversibly affect their structure and function, a phenomenon commonly known as nitrosative (or nitroxidative) stress [9]. Tyrosine nitration is one of the modifications occurring under conditions of NO overproduction and mostly depends on the reaction with ONOO^−^ [10]. It consists of a covalent addition of a nitro group (-NO_2_) to one of the two equivalent orthocarbons of the aromatic ring in tyrosine residues [11]. Although there are indications arguing for the existence of a denitrase activity, this has been not well characterized yet, and tyrosine nitration is still considered an irreversible modification of proteins subjected to massive nitroxidative stress. Indeed, elevated levels of tyrosine-nitrated proteins are reported in several neurodegenerative diseases and are commonly used as pathological markers of nitrosative stress [12–14].

### 1.1. *S*-Nitrosylation

**Figure 1:**
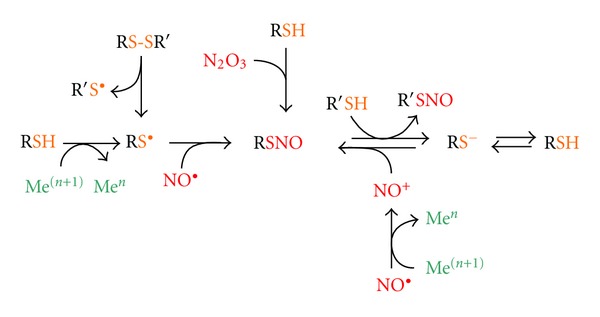
Mechanisms of *S*-nitrosylation. Cysteines of low molecular weight (e.g., GSH) and protein sulfhydryls (both termed as RSH) can undergo *S*-nitrosylation, thus generating *S*-nitrosothiols (RSNO), by different reactions involving different NO groups and different thiol substrates. RSNO can be formed upon the encountering of NO^•^ with a thiyl radical (RS^•^), with the latter species deriving from an RSH upon metal-catalyzed oxidation or upon homolytic scission of a disulfide bridge (RS-SR′) (on the left). However, as SH^•^ is a rare and chemically unstable species, it is plausible to consider that the majority of cellular RSNO generates from the thiolate form of the cysteine (RS^−^) that can result from sulfur deprotonation even at physiological pH. Either as RS^−^, or directly as RSH, cysteine sulfhydryl can undergo nitrosylation by reacting with NO-derived dinitrogen trioxide (N_2_O_3_), or directly with nitrosonium ion (NO^+^) generated upon metal-catalyzed oxidation of NO^•^. The net transfer of NO^+^ from an RSNO to an R′S^−^ (transnitrosylation) also occurs inside the cells and represents a further reaction to produce *S*-nitrosylated adducts (on the right).

Besides these deleterious and pathological effects, NO and other RNS can also concur to modulate signal transduction upon certain stimuli by means of other mechanisms that lead to transient protein modification. The main chemical reaction underlying this mechanisms is the *S*-nitrosylation (or *S*-nitrosation) of cysteine residues [15] (Figure 1). It consists on the covalent addition of an NO moiety to a reactive sulfhydryl, which results in the formation of an *S*-nitrosothiol derivative (SNO). SNOs generation depends on several factors, such as the environmental hydrophobicity conditions, the net charge and hindrance of the microenvironment in which reactive cysteines are embedded, and the presence of oxygen. NO can directly produce SNO if thiol residue, which is going to be modified, is present under the form of thiyl radical (-S^•^) (Figure 1). Nevertheless, this is a rare and unstable species; therefore, it is reasonable that the large amount of cellular SNOs generates from the reaction of thiols (present or not as thiolate anion, -S^−^) with the NO-derived species dinitrogen trioxide (N_2_O_3_) or, directly, with nitrosonium ion (NO^+^). The NO^+^ group is directly transferable between different SNOs, by means of a process known as transnitrosation or transnitrosylation [16] (Figure 1). Due to its feature of specificity and reversibility, *S*-nitrosylation of reactive cysteines is a prototype mechanism of redox-based signaling [17].

### 1.2. Thiol-Based Redox Modifications and Denitrosylating Enzymes

**Figure 2:**
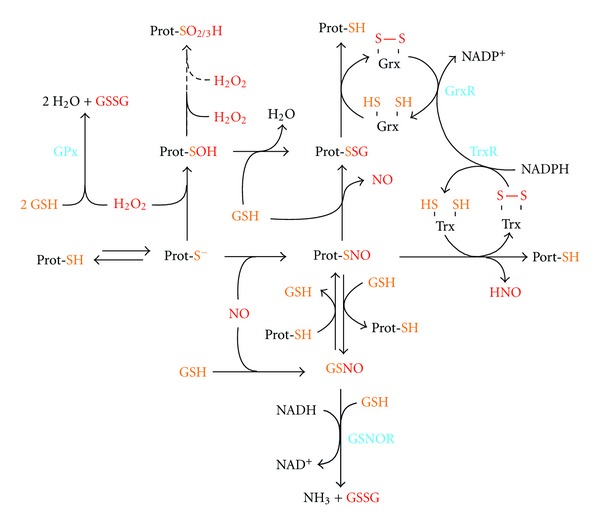
Redox network underlying protein thiol-dependent signaling. The key role of sulfur chemistry in cell signaling depends on the capability of specific cysteine residues, named *reactive cysteines*, of redox sensing proteins (Prot-SH) to undergo reversible oxidations upon deprotonation (formation of a thiolate adduct, Prot-S^−^). The net negative charge enhances the nucleophilic nature of sulfur and allows the generation of several adducts upon reaction with prooxidant compounds (red-colored). In particular, the encounter of a Prot-S^−^ with H_2_O_2_ leads to the hydroxylation of the sulfur moiety with the formation of a still reducible sulfenate derivative (SOH). Further H_2_O_2_-mediated oxidations modify sulfur to sulfinic (SO_2_H) or sulfonic (SO_3_H) acid species, that are irreversible oxidations, except for the former, that, in some cases (e.g., the sulfinic form of peroxiredoxin), can be reduced back at the expense of ATP by means of sulfiredoxin-mediated catalysis (not shown in the figure). Prot-S^−^ can also undergo *S*-nitrosylation, thereby generating a Prot-SNO adduct (see Figure 1). Both Prot-SNO and Prot-SOH can exchange with reduced glutathione (GSH), leading to the formation of the more stable *S*-glutathionylated species (Prot-SSG). Prot-SSG and Prot-SNO are reduced back, respectively, by the glutaredoxin/glutaredoxin reductase (Grx/GrxR) and thioredoxin/thioredoxin reductase (Trx/TrxR) systems, at the expense of NADPH. In addition, Prot-SNO can undergo transnitrosylation reactions with GSH, thereby forming *S*-nitrosoglutathione (GSNO). This reaction underlies a delicate equilibrium between the redox couples GSH/GSNO and Prot-SH/Prot-SNO that are strictly maintained by GSNO reductase (GSNOR) activity. Indeed, by using GSH-provided reducing equivalents and NADH as cofactor, GSNOR completely reduces GSNO to glutathione disulfide (GSSG) and ammonia (NH_3_), thereby deeply affecting Prot-SNO concentration. Intracellular GSH availability is also important to detoxify from H_2_O_2_ toxicity, as it is the elective cofactor of glutathione peroxidase (GPx). Therefore, GPx and GSNOR indirectly impact (i.e., *via* GSH oxidation to GSSG) on the total level of the reversibly oxidized proteins (Prot-SOH and Prot-SNO), by directly regulating the concentration of H_2_O_2_ and GSNO.

Similarly to cysteine sulfenate derivative (-SOH, see Figure 2), SNOs are relatively unstable adducts that can undergo exchange reactions with reduced glutathione (GSH) to generate more stable *S*-glutathionylated (-SSG) species, or, as demonstrated for matrix metalloproteinases, be further oxidized to sulfinate (-SO_2_H) or sulfonate (-SO_3_H) derivatives [18]. On the other hand, SNOs can be reduced back to sulfhydryl state by denitrosylation reactions [19]. More properly, SNO to SH conversion takes place by means of transnitrosylation reactions with a further cellular thiol moiety, the most representative of which are the low-molecular-weight antioxidant glutathione (GSH) and dithiol-containing oxidoreductases (Figure 2). Among this class of enzymes, thioredoxins (Trxs) are the best characterized examples of denitrosylases [20, 21], although other proteins, such as protein disulfide isomerase and glutathione-*S*-transferase *π*, have been suggested to act in the same way [19]. Trx-mediated reduction of SNOs leaves the NO moiety free being released intracellularly as nitroxyl (HNO) or NO, and Trx-contained dithiol being oxidized to disulfide bridge, which can be fully reduced to sulfhydryl state by the NADPH-dependent activity of the selenoprotein Trx reductase (TrxR) (Figure 2). This mechanism of denitrosylation has been largely described to influence the levels of protein SNOs; however, low-molecular-weight SNOs, such *S*-nitrosoglutathione (GSNO), can also undergo the same reaction [19]. Nevertheless, a direct NADH-dependent GSNO targeting enzyme, named GSNO reductase (GSNOR), has been discovered one decade ago and found to deeply impact on protein SNOs levels as well [22]. Due to mere chemical transnitrosylation reactions, indeed, the redox couples GSH/GSNO and protein-SH/protein-SNOs are in a dynamic equilibrium (Figure 2) therefore, by directly reducing GSNO, GSNOR indirectly decreases the concentration of protein SNOs. Actually, GSNOR is not properly a “new” enzyme, as it was one of the first enzymes to be discovered and characterized as the class III alcohol dehydrogenase (ADH III) or GSH-dependent formaldehyde dehydrogenase. However, in 1998, Jensen and coworkers found that GSNO is the elective substrate of ADH III, as the specific dehydrogenase activity was about the 6% of the GSNO reducing one [23]. Although both act as “SNO-scavenging” enzymes, Trx and GSNOR produce different side effects, which could differently affect cellular redox homeostasis. Indeed, whereas Trx-mediated denitrosylation leaves NO moiety being still reactive and available in forming adducts with proteins, GSNOR, by using GSH as cofactor, completely reduces NO to ammonia (NH_3_) and glutathione disulfide (GSSG), reason for which GSNOR has been also named “GSNO terminase.” Therefore, whereas NO is uniquely generated by NOS (except for the amount generated by the cytochrome *c* oxidase-mediated reduction of NO_2_^−^, the so-called “biology of nitrite anion,” see the following), there are at least two major enzymatic systems designed for removing NO group from *S*-nitrosylated cysteine thiol side chains: GSH/GSNOR and Trx/TrxR systems [19, 23] (Figure 2). The temporal and spatial regulation of production/removal of SNOs, as well as the diverse ability of Trx and GSNOR in denitrosylating SNOs, confers specificity to the NO-based cellular signaling [19, 22, 23].

This paper aims at describing the impact of nitrosylation/denitrosylation dynamics in mitochondrial function. In particular, the principal lines of evidence demonstrating the involvement of *S*-nitrosylation processes in respiratory chain efficiency, ATP production, apoptosis, but mostly in mitochondrial turnover and selective removal will be examined as regulatory events upstream of cellular dysfunctions concurring to cancer development and neurodegeneration.

## 2. Impact of NO and *S*-Nitrosylation Processes on Mitochondrial Homeostasis and Functions

### 2.1. Electron Transfer Chain

**Figure 3:**
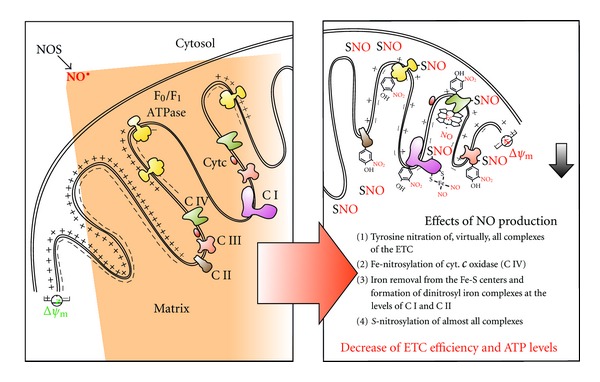
Effects of NO and nitrosative stress on mitochondrial electron transfer chain. NO, namely, the fraction produced in the cytosol by NOS, can cross cell membranes (e.g., the mitochondrial outer and inner membranes) and reversibly or irreversibly modifies mitochondrial complexes of the electron transfer chain (ETC). Specifically, nitration of all complexes, nitrosylation of Fe-heme-containing cytochrome *c* oxidase (C IV), generation of dinitrosyl iron complexes of Fe-S centers (e.g., those present in the Complex I), and *S*-nitrosylation of Complex I, III, IV, F_0_/F_1_ ATPase, as well as other unspecified mitochondrial proteins are shown. These modifications negatively affect ETC efficiency, and ATP production and decrease mitochondrial transmembrane potential (ΔΨ_m_), which represents e crucial event upstream of several mitochondrial functions, such as mitochondrial dynamics, mitophagy, and apoptosis.

Mitochondria accomplish a plethora of cellular functions, the best known of which is the oxidative phosphorylation, a process that ensures ATP neosynthesis in aerobic eukaryotes. During cell respiration, the electron flow generated through the respiratory chain is ultimately used for the tetravalent reduction of molecular oxygen at the level of cytochrome *c* oxidase. Concomitantly, ATP is synthesized by the F_0_/F_1_ ATP synthase exploiting the electrochemical proton gradient generated at the inner mitochondrial membrane. NO and RNS have been copiously reported to negatively affect mitochondrial respiration rate by inhibiting the activity of proteins implicated in this process, such as, virtually, all complexes of the electron transfer chain [24–26] (Figure 3). This inhibitory effect ranges from reversible to irreversible, up to be harmful for the entire mitochondrial compartment in dependence of (i) the concentration of NO and (ii) the RNS being engaged in the reactions. It is worthwhile noting, in fact, that mitochondria are the principal source of superoxide anion that can react at the diffusion-limited rate with NO to generate ONOO^−^. Therefore, the possibility that tyrosine nitration reactions could occur in metabolically active mitochondria is quite high. Indeed, all complexes have been demonstrated to undergo tyrosine nitration upon endogenous production of ONOO^−^ or after its administration [27].

NO itself, at physiological low (nanomolar) concentrations, can bind with high affinity to free Fe^2+^ or Fe^2+^ within any heme-containing protein with a free ligand position, such as cytochrome *c* oxidase, thus determining its inhibition [28] (Figure 3). In particular, NO reversibly binds to Fe^2+^ cytochrome a_3_ forming a nitrosyl-heme complex, condition that allows NO increasing the apparent K_m_ of cytochrome *c* oxidase for oxygen [29]. In such a way, even low physiological levels of NO can cause significant inhibition of respiration and potentially make it very sensitive to oxygen tension [30]. Since the reversible NO-mediated inhibition of cytochrome *c* oxidase occurs at nanomolar levels NO and in competition with oxygen [31], NO is considered a potential physiological regulator of respiration [32]. It is worthwhile noting that, besides competitive binding to Fe-heme, which remains the elective target of NO, and the main modification responsible for its inhibitory effects on mitochondrial respiration, NO has been reported to inhibit Complex IV activity also by binding the copper binuclear center of cytochrome *c* oxidase in a noncompetitive manner [33].

Similarly, NO can impact on mitochondrial respiration by reacting directly with iron of the iron-sulfur (Fe-S) centers of Complexes I and II, as well as aconitase (Figure 3) [25]. In this way, NO can damage iron-sulfur centers by removing iron (to form dinitrosyl iron complexes) and/or oxidize the iron-bound cysteine residues to disulfide or SNO. The formation of SNO derivatives can also occur on cysteine residues that are not engaged in the formation of iron-sulfur centers. Although in theory all complexes contain putative nitrosylable cysteines [25], the only evidence indicating how *S*-nitrosylation affects mitochondrial respiration deals with studies on Complexes I, IV, and ATPase (Figure 3). As in the case of other proteins, *S*-nitrosylation of mitochondrial complexes generally induces inhibition of protein function, thus reducing electron transfer and ATP production efficiency [25]. Particularly for what Complex I concerns, no comprehensive mechanism or specific cysteine residue undergoing *S*-nitrosylation has been reported so far, unless that the inhibition, which occurs at the 75 kDa subunit, is light-sensitive and reversed by reducing agents [32, 34, 35]. Studies of cardioprotection by GSNO also indicated that GSNO-preconditioned cardiomyocytes have a significant increase of *S*-nitrosylated F_1_ ATPase, *α*1 subunit, which causes a dose-dependent decrease of its activity [36]. In a more detailed manner, Zhang and colleagues found that, in lung endothelial cells, NO induces the selective *S*-nitrosylation of Cys^196^ and Cys^200^ residues of the mitochondrial Complex IV, subunit II, thereby allowing, also in this case, a transient inhibition of oxygen reduction [37].

ATP generation is coupled with the extrusion of H^+^from the mitochondrial matrix to the inner-membrane space, thus generating the proton motive force, which is used to drive the synthesis of ATP and other energy-requiring mitochondrial activities [38]. Proton motive force and the mitochondrial membrane potential (ΔΨ_m_) are then tightly related, so that ΔΨ_m_ represents a good indicator of the energy status of the mitochondrion and of the cellular homeostasis in general. The majority of the reports dealing with NO effects on mitochondrial homeostasis indicate that pathophysiological conditions in which NO is generated at high rate are tightly associated with mitochondrial membrane depolarization [26] (Figure 3). This event underlies several processes, such as mitochondrial dynamics, apoptosis, and autophagy.

### 2.2. Mitochondrial Dynamics

Mitochondria are in constant movement within cells, with fusion/fission events routinely taking place in order to allow physiological organelle turnover [39], to maximize mitochondrial efficiency [40], to regulate Ca^2+^ signaling/homeostasis and apoptotic response [41, 42], and to adapt ATP production to cellular energy demand [43]. Mitochondrial size, number, and mass are modulated by a variety of physiological stimuli. More than 1000 genes and ~20% of cellular proteins are involved in this process [42], and a complex regulatory network coordinates mitochondrial dynamics. Moreover, chemical species endogenously produced by the cell, such as NO, RNS, and ROS seem to play a key role in this process.

**Figure 4:**
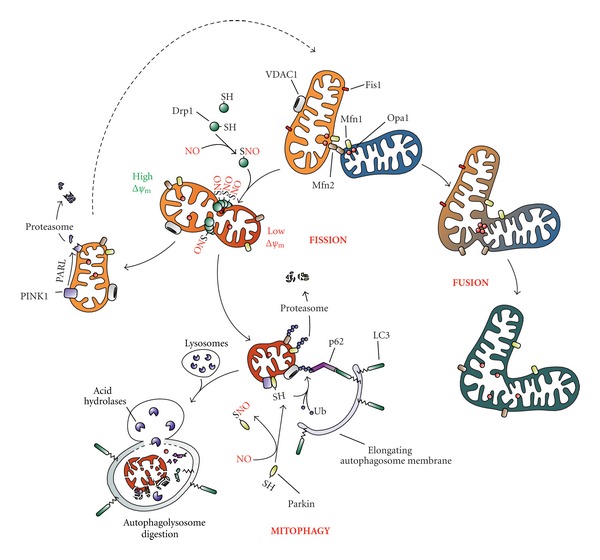
Effects of NO on mitochondrial dynamics and mitophagy. Mitochondrial network is dynamically regulated by fusion/fission events. Fusion between adjacent mitochondria (on the right) relies on the activity of Mfn1, Mfn2, and Opa1 which act in concert to mediate the merge of the outer and inner membrane, respectively. Although the presence of *S*-nitrosylated Opa1 has been observed, no role for this modification has been still proposed. Conversely, Drp1 has been reported to undergo several posttranslational modifications which modulate its fission activity (on the left), such as phosphorylation (not shown in the figure) and *S*-nitrosylation. Once *S*-nitrosylated and driven by mitochondria depolarization (low ΔΨ_m_), Drp1 is recruited onto the outer mitochondrial membrane by means of the recognition of its anchor protein Fis1. There, SNO-Drp1 multimerizes and acts to tighten the target organelle in order to share the depolarized portion from the healthy part. Although there is the possibility for a fragmented mitochondrion to refuse by means of Mfns and Opa1-mediated activity, frequently a depolarized organelle is targeted for its selective removal by autophagy (mitophagy). PINK1, which is normally degraded by PARL, is stabilized and recruits Parkin onto the outer membrane of an impaired mitochondrion and, in turn, catalyzes the covalent addition of an ubiquitin (Ub) tail to several protein targets. Ubiquitinated Mfns are extracted from the membrane and degraded *via* the proteasome in order to inhibit refusion processes, whereas ubiquitination of VDAC1 is required for mitochondria to be recognized and embedded by p62/LC3-bound autophagosome and ultimately degraded by lysosome-contained acid hydrolases. Parkin can undergo *S*-nitrosylation-mediated inactivation of its ubiquitin E3 ligase activity, thereby inhibiting mitophagy and disbalancing fusion/fission dynamics.

Mitochondrial fission contributes to the elimination of damaged mitochondrial fragments through mitochondrial autophagy (mitophagy) [44], whereas mitochondrial fusion facilitates the exchange of mitochondrial DNA (mtDNA) and metabolites needed for the maintenance of functional mitochondria [45] (Figure 4). Both events are controlled by four members of large GTPases: mitofusin 1 and 2 (Mfn1 and Mfn2), optic atrophy 1 (Opa1), and dynamin-related protein1 (Drp1), which are conserved from yeast to mammals, indicating that the fundamental mechanisms controlling mitochondrial dynamics have been maintained during evolution. Mfn1, Mfn2, and Opa1, act in concert to regulate mitochondrial fusion and cristae organization and localize in the outer and inner mitochondrial membrane [46], respectively, whereas Drp1 is a cytosolic protein, whose main function—that is induced upon translocation on the outer mitochondrial membrane—is to regulate mitochondrial fission [47] (Figure 4).

Mitochondrial fusion involves the tethering of two adjacent mitochondria followed by merging, or fusion, of the inner and outer mitochondrial membranes. Efficient mitochondrial fusion is important for cell viability as cells defective for fusion events display reduced cell growth, decreased ΔΨ_m_, and defective respiration [48]. In particular, studies on knockout mice have demonstrated the importance of Mfn1 and Mfn2 for mitochondrial fusion, as loss of both proteins leads to excessive mitochondrial fragmentation [49]. While Mfns are important for fusion of the outer mitochondrial membrane, Opa1 is pivotal for the fusion of inner mitochondrial membranes. Opa1 is a dynamin-related protein located on the mitochondrial inner membrane, and its ablation deeply impairs mitochondrial fusion [50]. Evidence also suggests that Opa1 has an important role in maintaining mitochondrial cristae structure, as loss of this protein results in disorganization of cristae and widening of cristae junctions [51].

During fission events, cytosol-distributed Drp1 localizes at the mitochondrial surface by means of Fis1, an integral outer mitochondrial membrane protein that interacts with Drp1 and functions as an exquisite mitochondrial Drp1 receptor [52]. Cells lacking Fis1 exhibit elongated mitochondria and a senescence-related phenotype, which lends the intriguing hypothesis that mitochondrial fission may counteract cellular senescence [53]. The putative relationship between mitochondrial dynamics and cell proliferation has been also reinforced by the identification that cell-cycle-dependent kinases phosphorylate and, thereby, modulate Drp1 activity [54].

Among the aforementioned large class of GTPases, Drp1 is the sole so far identified to be regulated by posttranslational modifications influencing its translocation onto the outer mitochondrial membrane and to induce mitochondrial fragmentation. For example, phosphorylation of several serine residues has been reported to modulate Drp1 activity [55], and the role (activating or inhibitory) of some of them still remains an issue of debate. However, it is well established that Cdk1/cyclin B-mediated phosphorylation of Ser^616^ activates Drp1 fission activity [56], whereas phosphorylation of Ser^637^ by cAMP-dependent protein kinase (PKA) is inhibitory [57]. In this regard, the calcium-dependent phosphatase calcineurin has been demonstrated to catalyze dephosphorylation of the same residue and to restore mitochondrial fragmentation process [58]. Sumoylation and *S*-nitrosylation have been reported to positively regulate Drp1-mediated mitochondrial fission as well. In particular, Cys^644^ has been identified to sense nitrosative stress. In accordance to Cho and coworkers [59], indeed, SNO-Drp1 translocates onto mitochondria and undergoes polymerization, which represents a structural modification stimulating GTP hydrolysis and allowing mitochondria to be fragmented (Figure 4). Consistent with these lines of evidence, C644A substitution of Drp1 abrogates fission events. In regard to these findings and their involvement in AD pathogenesis, the group of Bossy-Wetzel raised some concerns [60]. Indeed, though confirming that SNO-Drp1 represents a mitochondria-localized modification of the protein mainly present in postmortem brains from AD patients, the authors refuse that *S*-nitrosylation positively affects its enzymatic activity, leaving this issue still questionable. Interestingly, a significant amount of Opa1 was found to be *S*-nitrosylated in AD brain as well [60]; however, no implication for this modification in the regulation of mitochondrial dynamics has been never hypothesized.

### 2.3. Mitophagy

Autophagy is a self-degradation process activated by the cells under several pathophysiological conditions, such as nutrient deprivation, infection, development, and stressful conditions in general. It includes the chaperone mediated autophagy (CMA), microautophagy, and macroautophagy that are highly conserved degradation pathways for bulk cellular components [61, 62]. Macroautophagy (hereafter referred as to autophagy) is morphologically characterized by the formation of double-membrane autophagosomes, which sequester impaired or unwanted cellular components and deliver them to lysosomes for degradation and recycling of building blocks [62]. The mechanism of mitochondrial sequestration and delivery to lysosomes for degradation falls into this class and is commonly termed mitophagy [63, 64]. The elimination of mitochondria is a critical process as dysfunctional mitochondria produce higher amount of ROS which can be harmful for cellular biomolecules [65, 66]. However, under certain physiological conditions (e.g., erythroid differentiation, or starvation), mitophagy can also eliminate functional mitochondria [67, 68]. Mitochondrial depolarization is a hallmark of damaged mitochondria, and data from the recent literature argue for this being a prerequisite for mitophagy [69] (Figure 4). Two are the main proteins that are involved in targeting mitochondria to the selective removal by autophagy and whose mutations are associated with inherited forms of Parkinson's disease (PD): the PTEN-induced putative kinase 1 (PINK1) and the multifunctional ubiquitin E3 ligase Parkin.

#### 2.3.1. PINK1/Parkin System

Once synthesized, PINK1 is imported within mitochondria where undergoes cleavage catalyzed by the protease presenilin-associated rhomboid-like protein (PARL) in the mitochondrial inner membrane and then rapidly removed by a proteasome-dependent pathway [70] (Figure 4). Upon mitochondrial depolarization, PINK1 processing by PARL is inhibited, thereby leading to full-length PINK1 accumulation in the mitochondrial outer membrane, probably facing the cytosol [70, 71]. PINK1 stabilization is the driving event which leads to the recruitment of Parkin to mitochondria. In particular, mitochondrial-located Parkin promotes ubiquitylation of several protein substrates that are essential for the correct autophagosome targeting of mitochondria [70]. Indeed, once modified by ubiquitylation, a number of proteins (e.g., the voltage dependent anion channel 1, VDAC1) are recognized and bound by the ubiquitin-binding adaptor protein p62/SQSTM1 (p62), that concomitantly binds the autophagosome-located microtubule-associated protein light chain 3 (LC3) [72]. This “bridge-like” function of p62 lets fragmented mitochondria being correctly encompassed within the autophagosome without any possibility to re-fuse with the healthy mitochondrial network (Figure 4). This inhibition is guaranteed by the Parkin-mediated ubiquitylation of Mfn1 and Mfn2 that is a prerequisite for the extraction of both proteins from mitochondrial outer membrane through the catalytic activity of the AAA-type ATPase p97 and their subsequent degradation via the proteasome [73, 74] (Figure 4).

*S*-Nitrosylation is a well-established mechanism through which the ubiquitin E3 ligase activity of Parkin can be regulated [75] (Figure 4). At least five cysteine residues have been suggested to be potentially *S*-nitrosylated, thereby inhibiting Parkin activity [75]; however, for none of these the capability to undergo *S*-nitrosylation has been unequivocally reported. Very recently, Meng and coworkers have demonstrated that the formation of a sulfonic acid derivative at Cys^253^ induces Parkin aggregation and its incapability to translocate to mitochondria upon H_2_O_2_ overproduction, such as that occurring in PD-like conditions [76]. Although sulfonylation is an irreversible modification of the protein, it can be speculated that Cys^253^ could be particularly susceptible to oxidation by ROS, as well as by RNS, and that it could also react with NO, thus reversibly generating inactive SNO adducts of Parkin. Whatever is the residue involved in the generation of Parkin-SNO derivative, it is worthwhile mentioning that, thus modified, Parkin is no longer able to exert protective (antiapoptotic) effects in neuronal cell systems challenged with mitochondrial toxins or proteasome inhibitors. On the basis of what previously reported, it looks likely to hypothesize that *S*-nitrosylation of Parkin could negatively affect cell viability by impairing mitochondrial mitophagy. However, no direct evidence that Parkin-mediated protection of neuronal cells relies upon its capability to correctly induce mitochondrial degradation has been provided yet.

#### 2.3.2. HDAC6

It has been recently reported that, alongside p62, the class II histone deacetylase 6 (HDAC6) is even required for Parkin-mediated mitophagy and for perinuclear transport of depolarized mitochondria [77]. HDAC6 contains a nuclear exclusion signal and a cytoplasmic retention signal making it a cytoplasmic enzyme, whose main function is to catalyze tubulin deacetylation [78] and to play key regulatory roles in microtubule dynamics [79] and motor protein motility [80]. Although *S*-nitrosylation has been reported impairing the activity of cytosolic HDACs [81], no study has been performed aimed at comprehending whether it specifically targets HDAC6. Interestingly, HDAC6 is the main class II HDAC member reported to reside in the cytoplasm [82]; therefore, it lets presume that, effectively, HDAC6 could undergo *S*-nitrosylation. Nevertheless, direct evidence demonstrating the presence of its nitrosylated form is still lacking.

#### 2.3.3. DJ-1

Another protein that deserves to be mentioned in this context and whose mutations have been associated with the genetic forms of Parkinson's disease (PD) is the redox-sensitive chaperone DJ-1 [83]. Although its physical and functional association with PINK1 and Parkin is still controversial, it has been clearly arising that DJ-1 plays a crucial role in the correct fusion/fission events and processes targeting mitochondria for mitophagy, as DJ-1-null cell systems show significant alteration in both these processes [84, 85]. DJ-1 has been proposed to be active as dimer and preserves mitochondria from oxidative damage as it can directly react with ROS and RNS by means of reactive cysteine sulfhydryls. In particular, three cysteine residues have been identified to be redox-sensitive, with the Cys^106^ undergoing sulfi(o)nylation, and Cys^46^ and Cys^53^ being modified by *S*-nitrosylation. So far, no definitive role for these modifications has been provided; however, sulfinylation of Cys^106^ seems to be protective for mitochondria against prooxidant conditions [86], such as those occurring upon PD toxins administration. It has been also recently demonstrated that sulfinilated Cys^106^ plays a crucial role in cell survival against UV radiations, as it interacts with and stabilizes the antiapoptotic protein Bcl-X_L_, thereby preventing its degradation *via* the proteasome system [87]. On the contrary, since its first characterization [88], *S*-nitrosylation of DJ-1 has been implicated to allow the correct dimerization of the protein. In this regard, Cys^46^, but not Cys^53^—which is even nitrosylated—seems to assist DJ-1 dimerization, as C46A substitution is the sole mutation that completely abrogates the formation of DJ-1 dimers [88].

### 2.4. Apoptosis

**Figure 5:**
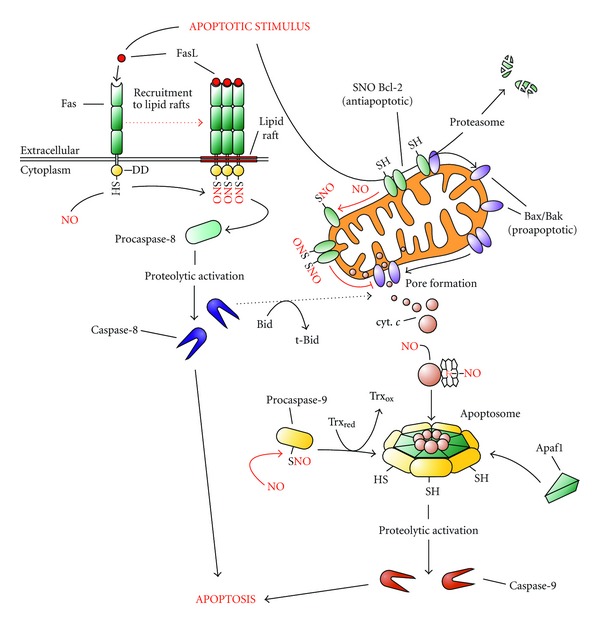
Effects of NO and nitrosative stress on apoptosis. NO-mediated effect on cell viability and death has been carefully characterized in the last years. For example, Bcl-2 has been reported to be *S*-nitrosylated and thus modified to be stabilized and not degraded by the Ubiquitin/proteasome system. Cytochrome *c *has been also indicated to undergo *S*-nitrosylation in order to bind Apaf1 and procaspase-9 and promote the assembling of the apoptosome. Zymogen procaspase-9, and the executioner pro-caspase3, remain in a quiescent (inactive) form since they are *S*-nitrosylated in their catalytic cysteine residue in order to avoid unwanted activation of death program. Upon apoptotic stimulus, Trxs are able to denitrosylate caspases, thereby allowing their proteolytic activation and the progression of the apoptotic events downstream it. Recently, it has been also highlighted that the recruitment of the death receptor Fas to lipid rafts of plasma membrane upon binding to its ligand (FasL) is enhanced by *S*-nitrosylation of Cys^304^ of its cytoplasmic domain (DD, death domain). In this case *S*-nitrosylation positively affects the execution of apoptosis that takes place directly *via* the caspase-8-initiated extrinsic route or can synergize with the mitochondrial pathway through the proteolytic activation of the proapoptotic protein Bid in its truncated form (t-Bid).

Apoptosis is a mode of programmed cell death that is crucial for mammalian development and whose deregulation may contribute to the development of neurodegenerative disorders and cancer [89]. Cells are routinely exposed to various stimuli that can be interpreted either as good or harmful and that determine whether downstream pathways should be transduced towards life or death. In several apoptotic pathways, such a choice is made at the level of mitochondria. These organelles are permeabilized by the proapoptotic proteins of the Bcl-2 family (e.g., Bax and Bak), that are generally antagonized by the antiapoptotic members of the same family (e.g., Bcl-2 itself, Bcl-X_L_), which mainly lead to the release of cytochrome *c* into the cytosol where it concurs to caspase activation and degradation of the entire cellular content (Figure 5) [90].

NO generated from NO donors, or synthesized by NOS, has been copiously demonstrated to induce cell death *via* apoptosis in a variety of different cell types; however, other pieces of evidence argue for NO being a protective molecule against proapoptotic stimuli [91]. The evidence to be, at the same time, pro- and antiapoptotic was found to depend on the concentration of NO employed, with nanomolar range inducing Akt phosphorylation and hypoxia inducible factor (HIF)-1*α* stabilization (prosurvival pathways), whereas micromolar levels triggering phosphorylation of p53 and the induction of apoptosis downstream of it [91]. This double feature confers to NO the name of “Janus-faced” molecule. Besides these effects which rely on the role of nitrosative stress as upstream inducer of signaling cascade, NO-mediated *S*-nitrosylation events have been reported to directly modulate a number of proteins involved in apoptotic response. Among them, cytochrome *c* should be undoubtedly mentioned, although it does not undergo *S*-nitrosylation. Indeed, NO binds the protein on its heme iron, in a way resembling the heme nitrosylation of cytochrome *c* oxidase, and this modification has been reported to occur during apoptosis and to positively influence the induction of cell death [92] (Figure 5). The release of cytochrome *c* from mitochondria is a crucial step in apoptosis, and, as above mentioned, it depends on the outer mitochondrial membrane amount of proapoptotic *versus* antiapoptotic members of the Bcl-2 superfamily. Regarding this, issue it should be reminded that Bcl-2 has been found to undergo *S*-nitrosylation at the level of Cys^158^ and Cys^229^ [93]. These modifications, that are not related to Bcl-2 phosphorylation, have been indicated to be crucial to stabilize the protein and to inhibit its degradation *via* the proteasome system, acting, in such a way, as an antiapoptotic event [93] (Figure 5). Generally, *S*-nitrosylation reactions are considered inhibitory of apoptotic cell demise. Indeed, many positive regulators of the apoptotic process, such as the L-type Ca^2+^  channel [25, 94] and the mitochondrial permeability transition pore components cyclophilin D [25, 95], ANT and VDAC [25, 96, 97] have been reported to undergo *S*-nitrosylation as protective mechanism against apoptosis. Although the list of proapoptotic members belonging to this redox-sensing class of proteins can be widely extended, *S*-nitrosylation of caspases remains the prototype of how this posttranslational modification can impact on the apoptotic signal [20, 98]. That *S*-nitrosylation was inhibitory for caspase proteolytic activity is a concept that goes back to the late 90s, where a number of publications showed that NO donors were able to inhibit apoptosis due to the occurrence of *S*-nitrosylation of cysteine-based enzymes involved in the execution of programmed cell death, such as caspase-3 and tissue transglutaminase [99], caspase-1 [100], and almost all caspases [101]. Afterwards, when Mannick and colleagues found that the sole mitochondrial subpopulation of caspase-9 and caspase-3, but not the cytosolic counterpart, were *S*-nitrosylated [98], the role of *S*-nitrosylation in the apoptotic context became clear. Mitochondria-generated NO leaves mitochondrial-located caspases in a quiescent state to inhibit unwanted activations of apoptosis but allows their induction whenever they are released in the cytosol downstream of an apoptotic stimulus. Accordingly, it was concomitantly found that Fas-induced apoptosis needs cytosolic caspases denitrosylation to proceed [102] (Figure 5). Although not directly involving mitochondria, a novel regulatory pathway that regulates apoptosis and that depends on *S*-nitrosylation has been reported to occur on the cytoplasmic domain of the death receptor Fas. In particular, Leon-Bollotte and coworkers demonstrated that both Cys^199^ and Cys^304^ of Fas intracellular portion undergo *S*-nitrosylation upon treatment with the NO donor glyceryl trinitrate, or the NOS activating molecule monophosphoryl lipid A, with the former thiol residue being indispensable for Fas recruitment to lipid drafts and activation of downstream apoptotic signal (Figure 5) [103].

## 3. Role and Mediators of Denitrosylation**** Process in Mitochondrial Homeostasis

NO can cross cell membranes. Therefore, once produced by NOS, it can freely pass mitochondrial membranes and act inside this organelle (Figure 3). In addition, some lines of evidence argue for the existence of a mitochondrial-sited isoform of NOS (mtNOS), that can directly regulate mitochondrial respiration and functions [104]. However, this aspect of NO biology remains still controversial as several studies let to hypothesize that the presence of any mitochondrial-associated NOS activity could be, merely, the consequence of experimental artifacts linked to mitochondrial purification [105]. In particular, this suspect takes cue from several observations indicating that mtNOS and nNOS are the same enzyme. Indeed, no canonical mitochondrial localization sequence, which could allow to discriminate between the cytosolic and the mitochondrial form of nNOS, has been never found. Apart from the possibility to be or not generated by a mitochondrial form of NOS, it should be reminded that, under hypoxic conditions, NO can be generated within the mitochondria without any NOS-dependent catalysis, but through the cytochrome *c* oxidase-mediated reduction of nitrite (NO_2_^−^) back to NO [106]. This body of evidence, though leaving questionable the precise site of production of mitochondrial NO (inside or outside the organelle), provides an indication about the high exposure/susceptibility of mitochondria towards nitrosative stress conditions. To have a general idea of how a mitochondria can suffer nitrosative stress, it should be taken into consideration that they are furnished by a large amount of mitochondrial-sited antioxidant and denitrosylating enzymes, which play a key role in modulating NO effect. Indeed, their scavenging activity counteracts the noxious effects of NO and decreases the effects of *S*-nitrosylation. Of note, the equilibrium between the opposite function of NO sources and systems aimed at mitigating NO effects is made more complex if one takes into account that many members of antioxidants enzymes and denitrosylases (e.g., glutathione reductase, glutathione peroxidase, peroxiredoxins, Trx, glutaredoxin 1) undergo thiol *S*-nitrosylation (or oxidation) that commonly results in the inhibition of their activity [107]. Letting this issue be omitted and focusing only on the contribution of denitrosylation reactions, it is worthwhile reminding that both the denitrosylating enzymes GSNOR and Trx1 have never been found to localize inside or to be associated with mitochondria; therefore, in theory, they cannot directly modulate mitochondrial SNOs levels. Moreover, no direct evidence has been provided yet in support of the sole mitochondrial form of thioredoxin (Trx2) being able to reduce *S*-nitrosylated complexes, or other mitochondrial proteins, and restoring electron transfer chain efficiency. Therefore, a question spontaneously arising is “what is the main denitrosylating enzyme implicated in the modulation of mitochondrial SNOs levels?” Recent observations arguing for a protective role of mitochondrial glutaredoxin 2 (Grx2) in *in vitro* models of neurodegeneration have been reported [108, 109]. As it is insensitive to *S*-nitrosylation [110] and has been demonstrated to catalyze reduction reactions of several *S*-glutathionylated mitochondrial proteins [109], a putative implication of Grx2 in mitochondrial denitrosylation reactions should be considered. Besides the putative roles of Trx2 and Grx2 as mitochondrial denitrosylating enzymes, it should be taken into account that GSH is present at high concentrations within the cell and that it can translocate in/out the mitochondrial membranes through the facilitative dicarboxylate transporters (DCTs). Given the capability of GSH to take part to transnitrosylation reactions with protein-SNOs, GSH is reasonably included among the principal candidates for denitrosylation of mitochondrial protein-SNOs. When GSNO is formed upon reaction of GSH with mitochondrial SNOs, it can be extruded in the cytosol by means of DCTs and there reduced by GSNOR, making this cytosolic enzyme the most reliable player of the complete reduction of mitochondrial *S*-nitrosylated proteome. This hypothesis is reinforced if one considers the large contribute that free GSH provides in denitrosylation of protein-SNOs from spinal cord challenged by exogenous supplementation of NO by means of transnitrosylation reactions [111].

On the basis of these assumptions, we can speculate that GSNOR deficiency or mutation could be predictive of mitochondrial morbidity towards nitrosative stress. Data from the recent literature demonstrate that GSNOR deficiency severely impacts on different aspects of mammalian physiology. For example, it (i) protects from heart failure and asthma [112, 113]; (ii) decreases vascular resistance [22, 114]; (iii) worsens septic shock conditions [114]; (iv) increases angiogenesis and protect against myocardial injury [115]; (v) compromises lymphocyte development [116]; (vi) weakens DNA damage response [117, 118]; however, no indication about whether some of these effects depend on *S*-nitrosylation-induced mitochondrial impairment has been provided so far. What is certain is that the last three effects of the above-mentioned list, alongside the observation that GSNOR is the sole alcohol dehydrogenase expressed in adult rat, mouse, and human brain [119], argue for a strong implication of GSNOR, and *S*-nitrosylation disbalance, in tumorigenesis and neurodegeneration.

## 4. Redox State and Energetic Metabolism**** in Cancer and Neuronal Cells: Role of Mitochondria and Possible Modulation by *S*-Nitrosylation

The mitochondrial theory of aging is based on the hypothesis of a vicious cycle, in which somatic mutations of mtDNA, such as those caused by ROS and RNS overproduction [120], generate respiratory chain dysfunction, thus enhancing the production of further DNA-damaging events. Therefore, chronic alterations of mitochondrial homeostasis, such as those impairing the removal of damaged (e.g., radical-producing) mitochondria, are a major event in the onset of several pathological states. Cancer and neurodegeneration, which are included in the list of “nitroxidative stress-based” diseases, are the two sides of the same molecular dysfunction. Indeed, if from one hand nitroxidative conditions can be deleterious for cell survival, as demonstrated by the massive cell death phenomena of neuronal populations observed during neurodegenerative processes, from the other one, they can induce mutagenesis and trigger limitless replication, condition occurring upon neoplastic transformation [121–123]. From a mere metabolic point of view, the maintenance of vital mitochondria and efficient oxidative phosphorylation is, in theory, a prerequisite much more critical for survival of neurons than for cancer cells. Indeed, tumor cells obtain ATP almost entirely by means of glycolysis even in normoxic conditions (the so-called Warburg effect or aerobic glycolysis), which represents a major change of the entire metabolic reprogramming typical of tumors [124, 125].

### 4.1. Role of *S*-Nitrosylation of HIF-1 in Tumor Metabolic Changes

Cancer cells have developed the aptitude to grow under low oxygen tension in order to face up the inability of local vessels to supply adequate amount of oxygen. Therefore, the upregulation of glycolytic pathway is a selective advantage to sustain ATP demand needed for tumor proliferation under hypoxic conditions. One of the major regulators of this metabolic change is HIF-1, a heterodimeric transcription factor composed of an oxygen-sensing *α* subunit and a constitutively expressed *β* subunit [126]. In normoxic conditions HIF-1*α* undergoes rapid proteasomal degradation elicited by prolyl-hydroxylases- (PHDs-) mediated hydroxylation, which lets HIF-1*α* being recognized for the subsequent ubiquitylation [127, 128]. Even under normoxic conditions, NO positively affects HIF-1 stabilization by indirectly inhibiting PHDs activity [129]; however, it has been also indicated that NO can directly impact on HIF-1*α* subunit by means of *S*-nitrosylation reactions on specific cysteines, thereby enhancing its stability and gene transactivating capacity [130]. In particular, Li and collaborators demonstrated that a specific *S*-nitrosylation event of HIF-1*α* on Cys^533^ inhibits its degradation as this modification stabilizes the protein, thereby determining the overall activity of HIF-1 [131]. Moreover, it has been also found that Cys^800^ located at the C-terminal activation domain can undergo *S*-nitrosylation and, thus modified, facilitates HIF-1 binding to its co-activator p300/CREB, thereby allowing the activation of HIF-1-mediated gene transcription. HIF-1 activation causes the induction of pyruvate dehydrogenase kinase 1 which shunts pyruvate away from mitochondria and concomitantly triggers mitophagy by means of the alternative pathway that relies upon the induction of Bcl-2/adenovirus E1B 19 kDa protein-interacting protein 3 (BNIP3) [132, 133]. Overall, these metabolic rearrangements lead to a reduction in mitochondrial mass and to an enhancement of the glycolytic flux, therefore reinforcing the hypothesis that *S*-nitrosylation of HIF-1*α* might be involved in metabolic changes occurring in cell malignant transformation.

### 4.2. Nrf2/Keap1 System in Cancer and Neuronal Cells

By contrast, neurons are “addicted” to ATP synthesized by mitochondria, whose efficiency does not depend upon glucose availability, but are continuously fueled by pyruvate deriving from glia-provided lactate in a Cori's cycle-like manner [134, 135]. Indeed, glucose taken up by neurons is mainly redirected to the pentose phosphate pathway allowing the generation of NADPH to sustain sulfhydryl reductive pathways (e.g., denitrosylation) and antioxidant response in general, which are indispensable for neuron survival [135, 136]. Indeed, in normal conditions, ROS levels are finely controlled by the transcriptional induction of many antioxidant systems which are predominantly regulated by the nuclear factor (erythroid-derived 2)-like 2 (Nrf2) [137]. In several tumor histotypes, a basal activation of this transcription factor is induced, and several somatic mutations have been demonstrated to destroy the interaction between Nrf2 and its physiological inhibitor Kelch-like ECH-associated protein 1 (Keap1), thereby promoting the persistent activation of the Nrf2-mediated antioxidant/detoxifying response and tumorigenesis [138, 139]. At the same time, several observations indicate that the activation of Nrf2 by nitroxidative insults is protective against conditions recapitulating neurodegenerative diseases. Recently, it has been shown that *S*-nitrosylation of Keap1—that, in some cases, can resolve in disulfide bridge formation [140]—in neuronal cells induces a persistent activation of Nrf2 signaling by allowing the dissociation of Nrf2-Keap1 heterodimer [141, 142].

### 4.3. NOS and GSNOR in Tumorigenesis

On the basis of these pieces of evidence, NO produced at moderately high rate, alongside with *S*-nitrosylation-induced impairment of respiratory chain, can be more dangerous for neurons than for cancer cells, where, conversely, it can promote tumor survival and malignancy by inducing further mutagenic events. In regard to this, all NOS isoforms have been detected in tumor cells from a wide range of isolates [143, 144]. Ambs and coworkers also demonstrated that an NO-mediated upregulation of vascular endothelial growth factor is related to increased xenograft vascularisation, indicating that NO generated by NOS promotes blood vessel formation, thereby enhancing the ability of tumor to indefinitely grow [145, 146]. iNOS has been also found being expressed in hepatocellular carcinoma (HCC) and is often increased in the hepatocytes of patients with chronic hepatitis and alcoholic cirrhosis that predispose to HCC [147–149]. In line with this assumption, it has been very recently demonstrated that GSNOR-KO mice, which are no longer able to denitrosylate SNOs *via* GSNOR activity, spontaneously develop HCC [117, 118, 150]. Wei and collaborators demonstrated that the mechanism underlying hepatocyte transformation involves the inactivation of the DNA repair system [118]. Particularly, GSNOR deficiency, or somatic loss-of-function mutations (e.g., deletion) in *GSNOR* gene, which have been found being associated with many cases of hepatic cirrhosis and chronic hepatitis B or C, induces *S*-nitrosylation of the DNA repair system member O^6^-alkylguanine-DNA alkyltransferase (AGT). Thus modified, AGT is degraded via the proteasome, thereby failing DNA damage being repaired and allowing mutations being established [118].

## 5. *S*-Nitrosylation and Cellular Quality Control Efficiency in Cancer and Neurodegeneration

NO, *S*-nitrosylation, and mitochondrial defects have long been regarded as contributors of the neurodegenerative processes [59, 151–153]; indeed, it has been demonstrated that PD, Alzheimers disease (AD), amyotrophic lateral sclerosis (ALS), and Huntingtons disease (HD) are characterized by an increase in nitrosative stress in neurons [154–156]. *S*-nitrosylation of Parkin, protein disulfide isomerase (PDI), peroxiredoxin 2 (Prx2), X-linked inhibitor of apoptosis (XIAP), and Drp1 has been implicated in stress-induced neuronal death [59, 157–159] and has been also observed in brains from patients with neurological disorders. Of note, these proteins are key players in the regulation of mitochondrial dynamics/autophagy (Parkin, Drp1), antioxidant and antiapoptotic response (Prx2, XIAP), correct protein folding (PDI), which represent the processes reported to be widely affected in neurodegenerative diseases. In particular, *S*-nitrosylation of PDI catalytic cysteines has been demonstrated to inhibit its enzymatic activity [160], thereby leading to the accumulation of polyubiquitylated proteins and, in turn, to endoplasmic reticulum stress [161]. Also, both peroxidatic and resolving cysteines of Prx2 (Cys^51^ and Cys^172^, resp.), as well as cysteines of XIAP located in its BIR domain, have been demonstrated to undergo *S*-nitrosylation. This posttranslational modification transiently inhibits isomerase and chaperone-like activities of Prx2 [158], as well as antiapoptotic function of XIAP, although it leaves unaltered its ubiquitin E3 ligase activity [159].

Taking into account the above-mentioned observations, it clearly arises that the efficiency of the systems deputed to cellular quality control is crucial for the homeostasis of cellular physiology [162–165]. DNA repair system, autophagic/mitophagic machinery, and the ubiquitin/proteasome system (UPS) ensure the maintenance of a correct equipment of biomolecules and organelles, whereas apoptosis guarantees the final elimination of cells whose vital functions are definitely compromised. A number of proteins and enzymes involved in DNA repair, autophagy, ubiquitylation and protein degradation, as well as in apoptosis are continuously subjected to nitroxidative modification (e.g., *S*-nitrosylation) which can compromise their correct activity. However, whereas loss-of-function modification of DNA repair systems and apoptotic proteins have much more severe repercussions on neoplastic transformation, the alterations of autophagy and UPS operation are harmful mostly for neuronal cell survival.

### 5.1. Modulatory Role of NO and *S*-Nitrosylation in Autophagy: Relevance in Cancer and Neurodegeneration

Autophagy and the UPS act synergistically to hydrolyze damaged proteins; actually, autophagy is regarded as a backup system to complement proteasomal degradation when it is overwhelmed or incapable of dealing with specific aggregated substrates [166]. This aspect gains increasingly value in neuronal homeostasis in which the presence of protein aggregates has been frequently associated with the etiology of the disease, and autophagy is being defined as antiapoptotic and anti-neurodegenerative process [167]. Remarkably, aggregates of proteins involved in neurodegenerative disease, such as *α*-synuclein or mutant huntingtin, have been identified as substrates for autophagy [168, 169]. The recent observation that NO, reasonably via *S*-nitrosylation, inhibits autophagy and that NOS inhibition enhances the clearance of autophagic substrates and reduces neurodegeneration in HD models [170] reinforces the hypothesis that nitrosative stress-mediated protein aggregation in neurodegenerative disorders may be, in part, due to the inhibition of autophagy. The reason according to which a correct autophagic flux is necessary to preserve neuronal viability does not exclude that it could be also implicated in tumorigenesis. Nevertheless, this issue is still controversial. Indeed, a growing body of evidence argues for autophagy being a crucial process in oncogenesis and in tumor progression [171–173]. The still uncharacterized autophagic mechanism leading to suppress tumorigenesis could depend on (i) the removal of nitrooxidatively damaged biomolecules; (ii) the degradation of specific organelles or proteins essential for cell growth [174–177]. By contrast, many observations show that autophagy activation enables tumor long-term survival (i.e., when apoptosis is defective) keeping cancer cells alive when limited angiogenesis leads to nutrient deprivation and hypoxia [178, 179]. From this perspective, one would expect that increased autophagy could promote solid tumors growth. It should be reminded that autophagy can suppress tumorigenesis by means of the elimination of p62 [180]. As previously described, p62 is an adaptor protein that targets damaged mitochondria and ubiquitylated proteins and that drives the correct autophagosome membrane formation being, at last, degraded during the process. Autophagy defects produce p62 accumulation, thereby causing persistent Nrf2 activation. In this regard, Komatsu and colleagues demonstrated that autophagy defects leading to p62 accumulation may induce nuclear translocation of Nrf2 through the interaction between Keap1 and p62 [181]. This novel regulation of Nrf2 provides a convincing evidence allowing to speculate that increased NO levels and *S*-nitrosylated proteins, by inhibiting autophagic flux, could function as protooncogenic also by impacting on such a mechanism.

### 5.2. Effects of an Altered Mitophagy in Neurodegeneration: Focus on Parkin and Drp1

Besides its role in damaged proteins removal, autophagy is principally implicated in the elimination of large cellular portions and organelles, such as in the case of mitophagy [182]. Mitochondrial dysfunction is a specific feature of almost all neurodegenerative diseases; however, the connection between mitophagy and neuropathology has been predominantly explored with respect to PD [183–185]. Several reports indicate that defective mitophagy, *via* a lack of mitochondria targeting due to mutated Parkin, may be to blame for much of the pathological phenotypes observed in PD [186]. As above described, *S*-nitrosylation inhibits Parkin ubiquitin E3 ligase activity and its protective function [187], resembling in such a way the alteration typical of PD-associated mutations [75, 187]. We above described that Parkin interacts with and polyubiquitinates Mfn1 and Mfn2, thereby promoting their degradation via the proteasome [188]. Accordingly, PD-related mutations in Parkin attenuate the occurrence of these processes and lead to excessive mitochondrial fusion. Based on these findings, Glauser and colleagues suggest that a close relationship between Parkin and mitochondrial dynamics exists and that mutations in the protein affect mitochondrial fission/fusion event, thus inducing neuronal death [188]. The demonstration that Mfns are ubiquitylated by Parkin provides a support for a link between Parkin ubiquitin E3 ligase activity and mitochondrial dynamics. An intriguing possibility is that PD-associated mutations that impair the Parkin-mediated ubiquitylation and degradation of Mfns may result in excessive mitochondrial fusion and impaired mitophagy, leading to an accumulation of damaged or dysfunctional mitochondria. The disruption of this dynamic equilibrium may herald cell injury or death and contribute to neurodegenerative disorders, as well as tumor development. Conversely, neurons from postmortem human AD brains [189] show excessive fission that results in abnormally small mitochondria with fragmented cristae [189, 190]. This phenotype has been indicated to be dependent on *S*-nitrosylation of Drp1 at Cys^644^ and excessive activation of its fission activity [59]. Accordingly, the exposure to oligomeric amyloid *β* (A*β*) peptide of cell culture models of AD has been reported to induce mitochondrial fragmentation as observed upon NO donors administration [191–194]. This phenotype is associated with synaptic damage and apoptotic cell death, thereby suggesting that the *S*-nitrosylation of Drp1 contributes to AD pathogenesis. Thus, denitrosylation of Drp1, such as by inducing GSNOR activity, may represent a potential new therapeutic target for protecting neurons and their synapses in sporadic AD.

## 6. Concluding Remarks

In this paper we have focused on the possible consequences of NO bioactivity, especially *S*-nitrosylation, on mitochondrial homeostasis, and we have reported how they can impact on cancer and neurodegenerative diseases. We have shown that many proteins involved in respiration, mitochondrial turnover, and apoptosis are subjected to *S*-nitrosylation, thereby modulating cellular response and the correct occurrence of several cellular functions. Whereas the implication of *S*-nitrosylation in apoptosis has been copiously investigated, research dealing with the role of redox-mediated posttranslational modifications in mitochondrial dynamics and mitophagy is still at the beginning phases. The comprehension of how redox mechanisms govern these phenomena will deserve deep investigations in the future in order to propose new pharmacological approaches able to interfere with *S*-nitrosylation state, and that can be useful as valuable tools for the treatment of diverse pathological conditions related to defect in mitochondrial dynamics and mitophagy. In regard to this aspect, great efforts in the last years have been profused by researchers to understand the involvement of the sole NO production in the onset of the diseases, without taking into account that SNOs levels can be modulated even by tuning denitrosylase activity. For instance, the observation that iNOS^−/−^ mice develop intestinal tumors led to substantiate the idea that iNOS was implicated in the macrophage-mediated tumor killing process [195]. In accordance, a growing body of evidence pointed out that NO-releasing drugs killed tumor cells [196–198]. However, the generation of NOS transfectants resulted in promotion of tumor growth, rather than killing, suggesting that, being NO a Janus-faced molecule, a precise modulation of its production rate is very hard to set up, and that even minor inaccuracies in setting the stage for clinical approaches could result in opposite effects [199, 200]. In line with these observations and consistent with the current opinion that NO is also, or rather principally, a molecule indispensible for cell viability and correct physiology, the putative use of the pan NOS inhibitor, N(G)-nitro-L-arginine methyl ester (L-NAME), has been reported to display a plethora of side effects that hamper its employment for the cure of aberrant *S*-nitrosylation-associated diseases [201]. At the mitochondrial level, for example, a reduction of NO levels, due to an inhibition of its production, profoundly affects the efficiency of oxidative phosphorylation and this could result in alterations of mitochondrial morphology and dynamics [202]. Results of the last years also argue for this relationship being biunivocal, as abnormal mitochondrial dynamics is strictly related to metabolic alterations [203]. Such an intimate interplay among processes involved in different aspects of mitochondrial homeostasis underlies the multiple mitochondrial phenotype observed in several neurodegenerative diseases. For example, patients with early stage AD, whose brains are distinguished by the presence of abnormal or fragmented mitochondria [189, 191], regularly exhibit declining mitochondrial energy metabolism and ATP production, which may subsequently cause synaptic loss and neuronal damage [203–205].

On the basis of what previously mentioned and taking into account that many proteins regulating mitochondrial dynamics and removal by autophagy are affected in their function by *S*-nitrosylation, it becomes clear that *S*-nitrosylation of this class of proteins could also impact on mitochondrial respiration and metabolism [206]. Indeed, Drp1 has been shown to be instrumental for sustaining mitochondrial ATP synthesis, as mitochondrial bioenergetics in Drp1-depleted cells is profoundly impaired [206, 207]. *Vice versa*, pharmacological inhibition of respiratory chain Complex I alters the organization of the mitochondrial network, which is paralleled by decreases in the mitochondrial membrane potential and an increased ROS production [206, 208]. In addition, the increased ROS production occurring under hyperglycemic condition requires dynamic changes in the morphology of mitochondria, with fragmentation being a necessary event to increase high-glucose-induced respiration and, in turn, to generate ROS [207]. Interestingly, cells expressing a dominant-negative mutant form of Drp1 show mitochondria that retain their tubular form and do not exhibit any increased respiration, hyperpolarization, or ROS production.

Altogether, these indications argue for regulation of mitochondrial morphology being intimately associated with the metabolic function of the organelle [207]; however, the molecular nature of this link remains still unknown. Animal models in which the denitrosylase activity is genetically impaired (e.g., GSNOR-KO mice) have been very recently employed to characterize the involvement of aberrant *S*-nitrosylation in liver cancer development. Nevertheless, the finding that AGT *S*-nitrosylation is a driving event in HCC does not exclude that other mechanism(s) could be operative for neoplastic transformation. Indeed, AGT-KO mice do not recapitulate GSNOR-KO phenotype, as they do not necessarily develop HCC [117]. On the basis of what has been described in this paper, it is reasonable to hypothesize that other factors related to mitochondrial homeostasis maintenance may have a role in the etiopathogenesis of liver cancer. It is desirable that, in the next future, transgenic mouse models of impaired denitrosylation, alongside with synthetic inhibitors of denitrosylating enzymes, namely, GSNOR, which have been recently designed [209–211] and yielded for the treatment of asthma, will be used to dissect how denitrosylation reactions, mainly those occurring within the mitochondria, are involved in the onset of several diseases. The results originating from these studies will provide the proof-of-principle of how, and whether, *S*-nitrosothiols targeting, *via* denitrosylase inhibition, could be a promising tool for the treatment of cancer and neurodegeneration.

## List of Abbreviations

AD: Alzheimers disease
ADH III: Class III alcohol dehydrogenase
AGT: O^6^-alkylguanine-DNA alkyltransferase
ALS: Amyotrophic lateral sclerosis
PKA: cAMP-dependent protein kinase
CMA: Chaperone mediated autophagy
DCTs: Dicarboxylate transporters
Drp1: Dynamin-related protein1
Grx: Glutaredoxin
GSH: Reduced glutathione
GSNO: *S*-Nitrosoglutathione
GSNOR: *S*-Nitrosoglutathione reductase
GSSG: Glutathione disulfide
HCC: Hepatocellular carcinoma
HD: Huntingtons disease
HDAC: Histone deacetylase
HIF1: Hypoxia inducible factor 1
Keap1: Kelch-like ECH-associated protein 1
L-NAME: N(G)-Nitro-L-arginine methyl ester
Mfn: Mitofusin
NO: Nitric oxide
NOS: Nitric oxide synthase
Nrf2: Nuclear factor (erythroid-derived 2)-like 2
ONOO^−^: Peroxynitrite
Opa1: Optic atrophy 1
PARL: Presenilin-associated rhomboid-like protein
PD: Parkinson's disease
PDI: Protein disulfide isomerase
PHDs: Prolyl hydroxylases
PINK1: PTEN-induced putative kinase 1
Prx2: Peroxiredoxin 2
RNS: Reactive nitrogen species
ROS: Reactive oxygen species
SNOs: *S*-Nitrosothiols
Trx: Thioredoxin
TrxR: Thioredoxin reductase
UPS: Ubiquitin/proteasome system
VDAC1: Voltage dependent anion channel 1
XIAP: X-linked inhibitor of apoptosis.
